# Fermented Biomass of *Arthrospira platensis* as a Potential Food Ingredient

**DOI:** 10.3390/antiox11020216

**Published:** 2022-01-23

**Authors:** Polona Jamnik, Nik Mahnič, Aleksandra Mrak, Lea Pogačnik, Barbara Jeršek, Alberto Niccolai, Jasmina Masten Rutar, Nives Ogrinc, Larisa Dušak, Blaž Ferjančič, Mojca Korošec, Ana Cerar, Borut Lazar, Urša Lovše, Tjaša Pungert, Primož Fabjan, Nataša Poklar Ulrih

**Affiliations:** 1 Department of Food Science and Technology, Biotechnical Faculty, University of Ljubljana, Jamnikarjeva 101, 1000 Ljubljana, Slovenia; nik.mahnic@bf.uni-lj.si (N.M.); aleksandraamrak@gmail.com (A.M.); lea.pogacnik@bf.uni-lj.si (L.P.); barbara.jersek@bf.uni-lj.si (B.J.); dusak.larisa@gmail.com (L.D.); blaz.ferjancic@bf.uni-lj.si (B.F.); mojca.korosec@bf.uni-lj.si (M.K.); ulovse@gmail.com (U.L.); tjaska.pungertova@gmail.com (T.P.); fabjan.primoz@gmail.com (P.F.); natasa.poklar@bf.uni-lj.si (N.P.U.); 2 Department of Agriculture, Food, Environment and Forestry (DAGRI), University of Florence, Piazzale delle Cascine 18, 50144 Florence, Italy; alberto.niccolai@unifi.it; 3 Department of Environmental Sciences, Jožef Stefan Institute, Jamova 39, 1000 Ljubljana, Slovenia; jasmina.masten@gmail.com (J.M.R.); nives.ogrinc@ijs.si (N.O.); 4 Jožef Stefan International Postgraduate School, Jamova 39, 1000 Ljubljana, Slovenia; 5 AlgEn, d.o.o., Brnčičeva Ulica 29, 1000 Ljubljana, Slovenia; ana@algen.si (A.C.); borut@algen.si (B.L.)

**Keywords:** *Arthrospira platensis*, lactic acid fermentation, *Lactobacillus plantarum*, antioxidant activity, microbiological safety, nutritional composition

## Abstract

Lactic acid fermentation (LAF) is known to improve nutritional properties and functionality and to extend the shelf life of foods. We studied the LAF of *Arthrospira platensis* as the sole substrate using *Lactobacillus plantarum* as the starter culture. Fermented (FB) and non-fermented broth (NFB) were analysed by means of pH, lactic acid bacteria (LAB) count, lactic acid concentration, microbiological safety, and nutritional composition. Additionally, water and ethanol extracts were prepared on which total phenolic content, DPPH radical scavenging activity, and cellular antioxidant activity were determined. The maximum increase in LAB count and lactic acid concentration and drop in pH was observed in the first 24 h of fermentation. Total phenolic content and DPPH radical scavinging activity of ethanol extracts increased after fermentation compared with NFB. Ethanol extracts of FB have been shown as a potential source of antioxidants, which efficiently lowered oxidation level in the cells of yeast *Saccharomyces cerevisiae*, as well as the oxidative damage of lipids. Additionally, the level of non-protein nitrogen increased, indicating higher protein bioavailability, and fat content decreased in comparison with NFB. No presence of pathogenic bacteria and low pH indicate enhancement of FB microbiological stability. Therefore, inclusion of fermented *A. platensis* into food products could lead to added-value foods based on microalgae.

## 1. Introduction

Lactic acid fermentation is known to improve the nutritional properties and functional value of food substrates and to enhance their shelf life and microbiological safety, as well as enhancing their sensory characteristics [1]. Both, the profile and amount of bioactive compounds is changed. Some molecules (bioactive peptides, polysaccharides, short-chain fatty acids) are generated, while antinutritional compounds and sugar content are decreased. Additionally, molecules with added biological value are generated after conversion of phenolic compounds. These transformations lead to improvements in the bioaccessibility and bioavailability of the food components, which is related to modification of their health-related properties [2]. As *Arthrospira platensis* (*Spirulina*) contains many functional bioactive constituents (e.g., long-chain polyunsaturated fatty acids, phenolic compounds, sterols, proteins, peptides, amino acids, vitamins, polysaccharides, pigments) with different activities [3], lactic acid fermentation might allow the preparation of food products based on microalgae with better nutritional and functional characteristics compared with the original non-fermented microalgal biomass. Uchida and Meyoshi [4] have already reported on lactic acid fermentation of microalgae (i.e., *Chlorella* spp., *Tetraselmis* spp., *Pavlova lutheri*, *Chaetoceros* spp., *Nannochloropsis* spp.) as well as macroalgae (i.e., seaweeds), and their studies have opened up the possibility of producing fermented foods from algae. A few studies have investigated *Spirulina* as a sole substrate for lactic acid fermentation. Niccolai et al. [5] investigated the use of lactic acid fermentation of *Spirulina* biomass as the sole substrate for the production of probiotic-based products. Their data showed that after 48 h of fermentation the concentration of *Lactobacillus plantarum* and lactic acid increased significantly. Additionally, the antioxidant activities *in vitro*, the total phenolic content, and digestibility increased after fermentation. Similarly, de Marco Castro et al. [6] established that the total phenolic content and antioxidant activity in vitro enhanced in fermented spirulina compared with untreated biomass. Additionally, protein fragmentation and free methionine content increased linearly with the fermentation time. Yu et al. [7] used three kinds of probiotic combinations (lactic acid bacteria, *Bacillus* strains, and their mixture) and showed different effects on *Spirulina* fermentation, in which the lactic acid bacteria and *Bacillus* strains showed positive effects in the context of flavour, nutrition, or bioactivity. Enhanced total antioxidant capacity and beta-carotene profile of *A. maxima* fermented by *L. plantarum* was thought to contribute to the apparent higher brain-derived neuroprotective factor compared with its untreated control [8]. Lactic acid fermentation of *Spirulina* was investigated also in terms of its use for cosmetic products, and it was shown that antioxidant, anti-inflammatory, and UV protective activities of fermented *Spirulina* increased compared with native *Spirulina* [9]. Higher levels of free polyphenols and phycocyanobilin were detected in fermented compared with non-fermented spirulina.

*L. plantarum* is extensively used as a starter culture as well as a probiotic microorganism in the food industry. The long history of *L. plantarum* strains application in food fermentation led to the design of added-value foods with improved nutritional and technological features [10].

To the best of our knowledge, no studies were found to compare *A. platensis* biomass as a sole substrate before and after fermentation with *L. plantarum* in the context of whole nutritional composition and energy value, microbiological safety, antioxidative activity in the cells, and lipid peroxidation. Additionally, two different extraction solvents were used to distinguish between polar and less polar antioxidants and to study the effect of lactic acid fermentation on their activity.

## 2. Materials and Methods

### 2.1. Lactobacillus plantarum Inoculum Preparation

*Lactobacillus plantarum* (LMG 6907) was obtained from the Institute of Dairy Science and Probiotics, Department of Animal Science, Biotechnical Faculty. A stock culture in 20% (*v/v*) glycerol was transferred into 20 mL of MRS broth (Merck, Darmstadt, Germany). Then overnight cultivation was performed on a rotary shaker (30 °C, 150 rpm). The overnight culture was centrifuged at 14,000× *g* 5 min and washed once with the physiological solution (0.9% (*w/v*) NaCl) to prepare the suspension for inoculation.

### 2.2. Arthrospira platensis Cultivation

Fresh *Arthrospira platensis* biomass was obtained from Severino Becagli algae farm (Grosseto, Italy) in collaboration with AlgEn, where it was cultivated in 500 m^2^ ponds under controlled conditions (pH 10.6). Constant mixing was ensured by a paddle wheel. Greenhouses with nets prevented the access of insects to the ponds. The quality of the *A. platensis* was ensured by high-tech processes with critical control points in all phases of production.

### 2.3. Lactic Acid Fermentation

An amount of 10 g of fresh *A. platensis* biomass was mixed with 10 mL of physiological solution to get a broth (non-fermented broth–NFB) sampled immediately after preparation. NFB was inoculated with *L. plantarum* suspension (1% (*v/v*) inoculum), and the fermentation was carried out at 30 °C for 72 h. Samples of fermented broth (FB) were taken at t = 0 (immediately after inoculation), 24, 48, and 72 h. Samples were frozen for further analyses, except for microbiological analysis and *L. plantarum* growth determination, where samples were analysed immediately after sampling.

### 2.4. L. plantarum Growth Determination

Non-fermented and fermented (t = 0, 24, 48, and 72 h) broth was diluted according to Koch and appropriate dilutions were transferred on MRS agar (Merck, Darmstadt, Germany) containing cycloheximide (Sigma-Aldrich, St. Louis, MO, USA) in a concentration of 100 mg/L. Plates with inoculated MRSc agar were incubated in an anaerobic jar at 30 °C, 48 h. After incubation, the number of colonies was counted, and results are expressed as logarithm of the number of colony-forming units (CFU) per g of broth-log CFU/g.

### 2.5. Determination of Lactic Acid Concentration

Samples were collected from NFB and FB (t = 0, 24, 48, 72 h), diluted with water (1:1), and centrifuged at 4000× *g* for 15 min at 4 °C. The supernatant was then incubated at 90 °C for 10 min to denature proteins, centrifuged at 12,000× *g* for 10 min, and then used for lactic acid determination according to the method of Borshchevskaya et al. [11]. An amount of 25 µL of supernatant was mixed with 1 mL of 0.2% (*w/v*) FeCl_3_ (FeCl_3_, Sigma-Aldrich, St. Louis, MO, USA), and then absorbance at 390 nm was measured. The concentration of lactic acid was obtained from a calibration curve using lactic acid as standard (Sigma-Aldrich, St. Louis, MO, USA) and expressed as g lactic acid/L. Additionally, at each time point pH value was measured.

### 2.6. Determination of Nutritional Composition

The chemical composition of microalgae biomass before and after lactic acid fermentation was performed to determine the key changes in certain macronutrient contents arising from the fermentation. Total and non-protein nitrogen were determined by the Kjeldahl method (AOAC 981.10) [12]. The calculated difference on behalf of proteinic nitrogen was multiplied by a general conversion factor 6.25 to evaluate the amount of crude protein. Crude fat content was determined by the Weibull–Stoldt method (AOAC 963.15), total mineral content by dry ashing at 550 °C (AOAC 920.181), and soluble and insoluble fraction of dietary fibre by the enzymatic–gravimetric method (AOAC 941.43) [12]. The available carbohydrate content was calculated as the difference between the dry mass, and the content of analysed nutrients and ash. Nutritional value was calculated using energy factors: 17 kJ/g for crude protein and available carbohydrate; 37 kJ/g for crude fat; and 8 kJ/g for total dietary fibre [12]. All results are reported per 100 g of broth dry weight.

### 2.7. Microbiological Analysis

The microbiological quality and safety of NFB and FB after 24 h of fermentation was determined by microbiological analysis (yeast and moulds (YM), aerobic mesophilic bacteria (AMB), anaerobic mesophilic bacteria (ANMB), aerobic spore-forming bacteria (ASFB), anaerobic spore-forming bacteria (ANSFB), lactic acid bacteria (LAB), coliform bacteria (CC), *Escherichia coli, Staphylococcus aureus*, *Bacillus cereus*, *Clostridium perfringens*, *Salmonella* spp., and *Listeria monocytogenes*). Samples were prepared for quantitative microbiological analysis by addition 10 g of broth to 90 mL of physiological solution (0.9% (*w/v*) NaCl) and homogenized (Stomacher, 1 min at medium speed). The number of yeast and moulds and the number of bacteria were determined with the plate count method with appropriate medium and incubation conditions. Media were dichloran rose-bengal chloramphenicol agar (Oxoid CM, Hampshire, UK) with chloramphenicol supplement (Oxoid, SR0078, Basingstoke, UK) for YM; plate count agar (Oxoid, Basingstoke, UK) for AMB and ANMB; thioglycollate agar (Oxoid, Basingstoke, UK) with 20 g/L of agar for ASFB and ANSFB; De Man, Rogosa, Sharpe medium (Oxoid, Basingstoke, UK) with cycloheximide (Sigma-Aldrich, St. Louis, MO, USA, 100 mg/L) for LAB; violet red bile lactose agar (Oxoid, Basingstoke, UK) for CC; tryptone bile x-glucuronide medium (Oxoid, Basingstoke, UK) for *E. coli*; Baird–Parker medium (Oxoid, Basingstoke, UK) with Egg Yolk Tellurite Emulsion (Oxoid) for *Staph. aureus*; *Bacillus cereus* agar (Oxoid, Basingstoke, UK) with polymyxin B supplement (Oxoid, SR0099) for *B. cereus*; Sulfite polymyxin sulfadizine agar (Merck, Darmstadt, Germany) for *Cl. perfringens*. *Salmonella* spp. and *Listeria monocytogenes* were analysed in 10 g of broth (NFB and FB) after non-selective enrichment of sample (10 g) in Universal pre-enrichment broth (UPB, Oxoid, Basingstoke, UK) and 24 h incubation of suspension at 37 °C, following isolation on Rambach agar (Merck) for *Salmonella* spp. and on ALOA medium (Oxoid, Basingstoke, UK) with Chromogenic *Listeria* Selective Supplement (Oxoid, SR0226, Basingstoke, UK) and ISO Differential Supplement (Oxoid, SR0244) for *L. monocytogenes*. For *L. monocytogenes,* Fraser broth (FB, Oxoid, Basingstoke, UK) with selective supplement (Oxoid, SR0156, Basingstoke, UK) as the second enrichment was used as well (0.1 mL of UPB was transferred to 10 mLFB, incubated 24–48 h), followed by isolation on ALOA medium. Agar plates for bacteria were incubated at 37 °C for 24–48 h, and agar plates for yeast and moulds were incubated at 25 °C for 5–7 days. For ANMB, ANSFB, and LAB agar plates were incubated under anaerobic conditions. After incubation, the number of typical colonies was counted, and results are expressed as logarithm of the average number of CFU per g of NFB or FB (log CFU/g).

### 2.8. Preparation of NFB and FB Extracts for Antioxidant Activity Determination

To determine the total phenolic content and antioxidant activity of *A. platensis* biomass before and after fermentation, extracts from NFB and FB were prepared using two different solvents: water and 96% ethanol.

To obtain higher yields, a two-stage extraction was performed. For the first stage, 8 g of NFB or FB was weighted into 50 mL centrifuge tubes, to which 12 mL of extraction solvent (water or 96% ethanol) was added. The extraction was performed for half an hour in a water bath (40 °C) with shaking. Afterwards, the samples were centrifuged for 10 min (6000 rpm), and the supernatant was collected. For the second stage, the remaining sediment was extracted with another 12 mL of each solvent following the same procedure. Both supernatants were joined to obtain the final extract, which was stored in a freezer at −20 °C until being analysed (determination of total phenolic content and antioxidant activity).

Concentrated extracts were used to determine antioxidant activity in the cells. The water extracts were freeze-dried, while the ethanol extracts were first evaporated and then freeze-dried in order to obtain dry extracts. The mass yields of water extracts were 11.5% (NFB) and 7.6% (FB), whereas for the ethanol extracts they were lower, 4.3% (NFB) and 5.6% (FB). The dry extracts were then dissolved in water for water extracts or DMSO (Sigma-Aldrich, St. Louis, MO, USA) for ethanol extracts in order to obtain concentrated extracts with a concentration of dry extract equal to 50 mg/mL (water extracts) and 100 mg/mL (ethanol extracts).

### 2.9. Total Phenolic Content

To determine total phenolic content Folin–Ciocalteu reagent was used followed by a spectrophotometric quantification [13,14]. Briefly, the reaction mixtures for calibration curves (for each solvent individually) were prepared with 25 to 200 μL of the standard solution of gallic acid (0.45 mM in water or 96% ethanol) and an adequate amount of the corresponding solvent to obtain 725 μL. To this, 125 μL of Folin–Ciocalteu reagent (freshly diluted in water; 1:2) was added, followed by the addition of 125 μL 20% Na_2_CO_3_ (in water) after exactly 5 min. After mixing, the samples were kept in the dark at ambient temperature for 60 min to finish the reaction, followed by absorbance measurement against a blank sample (water or 96% ethanol) at 765 nm. To determine the total phenolic content in extracts, 10 μL of water extracts and 40 μL of ethanol extracts were analysed the same way. The results are expressed as equivalent of gallic acid (in mg) per dry weight of broth (in g). Gallic acid and Na_2_CO_3_ were obtained from Sigma-Aldrich, St. Louis, MO, USA.

### 2.10. Antioxidant Activity In Vitro

Antioxidant activity in vitro was evaluated using the DPPH^•^ radical scavenging method [15]. Briefly, calibration curves for each solvent (water and 96% ethanol) were prepared as follows: 5 to 50 μL of Trolox standard solution (1.11 mM in water or 96% ethanol) and an adequate amount of the corresponding solvent to obtain 50 μL was mixed with freshly prepared 0.11 mM DPPH^•^ (in methanol). The absorbance of the reaction mixture was measured after one hour at 550 nm instead of the usually used wavelength 517 nm to avoid the interferences in the coloured extracts. To determine antioxidant activity in extracts, 35 μL of water extracts and 50 μL of ethanol extracts were analysed the same way. The results are expressed as Trolox equivalent antioxidant capacity (TEAC) in mg per dry weight of broth (in g). Trolox and DPPH^•^ were from Sigma-Aldrich, St. Louis, MO, USA.

### 2.11. Cellular Antioxidant Activity (CAA) Assay

Cellular antioxidant activity was evaluated by measuring intracellular oxidation in the yeast *Saccharomyces cerevisiae* as a model organism [16]. The yeast *S. cerevisiae* was provided from the Culture Collection of Industrial Microorganisms (Biotechnical Faculty, Slovenia). It was grown in YEPD medium (Sigma-Aldrich, St. Louis, MO, USA) at 28 °C and 220 rpm until the stationary phase, then the cells were washed and suspended in PBS buffer [17]. Yeast cells were treated with water and ethanol extracts of NFB and FB in a concentration of 1.5 mg and 3 mg dry extract/mL of yeast suspension for 2 h. Yeast cells treated with the same volume of solvent used were considered as controls. After treatment, the 2 mL of cell suspension were centrifuged (14,000× *g*, 5 min) and washed twice with 50 mM potassium phosphate buffer (pH 7.8). The cell pellets were resuspended in 0.99 mL of 50 mM potassium phosphate buffer. After a 5 min incubation at 28 °C, H_2_DCF diacetate (Sigma-Aldrich, St. Louis, MO, USA) was added to reach a final concentration of 10 µM and incubated for 30 min at 28 °C and 220 rpm. Then the fluorescence of the yeast suspension was measured by Safire II microplate reader (Tecan, Männedorf, Switzerland). The excitation wavelength of DCF was 488 nm, emission wavelength was 520 nm. The optical density of yeast suspension was measured at 650 nm.

Results are expressed as relative values of fluorescence/optical density to the corresponding control (yeast cells treated with water or DMSO).

### 2.12. Lipid Peroxidation

Yeast cells in the stationary phase were suspended in PBS buffer as described in Section 2.11. Cells were first exposed to ethanol extract of FB in the concentration of 1.5 mg dry extract/mL of yeast suspension for 2 h and then to 100 mM menadione for 1 h as an oxidative stress inductor. Control cells were exposed only to menadione (Sigma-Aldrich, St. Louis, MO, USA) without previous treatment with extracts. Lipid peroxidation was quantified by the determination of thiobarbituric acid (TBA)-reactive substances (TBARS) [18]. In both cases, cells were centrifuged (4000× *g*, 5 min) and washed once with PBS. To the sediment, a reagent containing 91.8 mM trichloroacetic acid (TCA, Merck, Darmstadt, Germany), 2.5 mM thiobarbituric acid (TBA, Merck, Darmstadt, Germany), 45.4 μM butylhydroxytoluene (BHT, Merck, Darmstadt, Germany), and 25 mM HCl (HCl, Merck, Darmstadt, Germany) was added. The cells were then disrupted by vortexing with 0.5 mm zirconia/silica beads (BioSpec Products, Inc., Bartlesville, OK, USA), twice for 4 min each time using a homogenizer (Bullet Blender Storm 24, Next Advance, Troy, New York, NY, USA) with 5 min interval for cooling the samples on ice. The cell homogenates were centrifuged at 13,000× *g* for 10 min. Supernatants were incubated at 90 °C (Thermomixer R, Eppendorf, Hamburg, Germany) for 30 min, and after cooling, 1-butanol was added. After centrifugation at 13,000× *g* for 10 min, 200 uL of upper butanolic phase was removed, and the fluorescence was measured, using a Varioskan™ LUX (Thermo Fisher, Waltham, MA, USA) microplate reader. The excitation and emission wavelengths were 515 nm and 555 nm, respectively. To normalize the fluorescence values, the optical density of the yeast suspension was measured at 650 nm. Results are expressed as relative values of fluorescence/optical density (F/OD) to the corresponding control.

### 2.13. Statistical Analysis

Fermentation of *A. platensis* biomass and consequently all analyses were conducted in at least three replicates. Data are expressed as means ± SD (standard deviation). Differences between non-fermented and fermented broths or treated and non-treated cells (cellular assays) were determined using the Student t-test and were considered statistically significant when *p* < 0.05.

## 3. Results

### 3.1. Lactic Acid Fermentation

**Figure 1:**
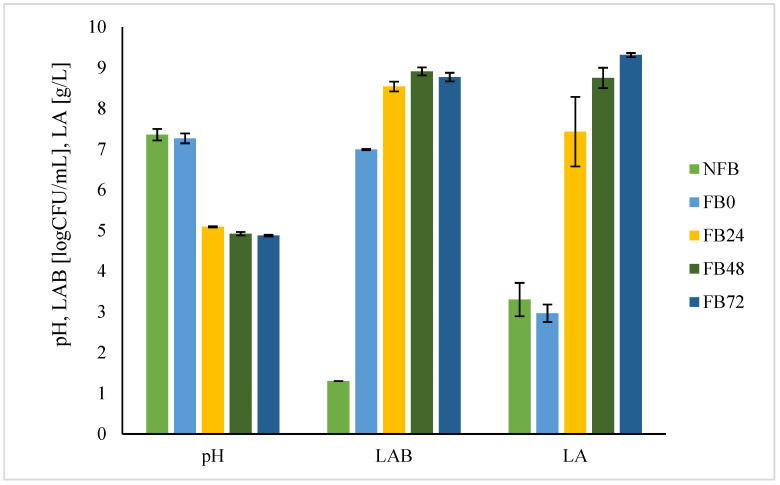
Determination of pH, LAB concentration (LAB), and lactic acid concentration (LA) in NFB and FB. Data represent mean values ± SD.

Figure 1 presents lactic acid fermentation of *A. platensis* broth inoculated with *L. plantarum*, where pH, lactic acid (LA), and LAB growth were followed.

The greatest changes were observed in the first 24 h. pH decreased from 7.3 to 5.1 and remained constant in the next 48 h. The growth of *L. plantarum* was the most rapid in the first 24 h. At inoculation (t = 0), the cultivability was 7.0 log CFU/g and increased to 8.5 log CFU/g after 24 h; later values remained the same. Lactic acid reached a concentration of 9.3 g/L at 72 h; again, the rapid increase was observed in the first 24 h, where values reached 7.4 g/L.

### 3.2. Microbiological Quality and Safety

**Figure 2:**
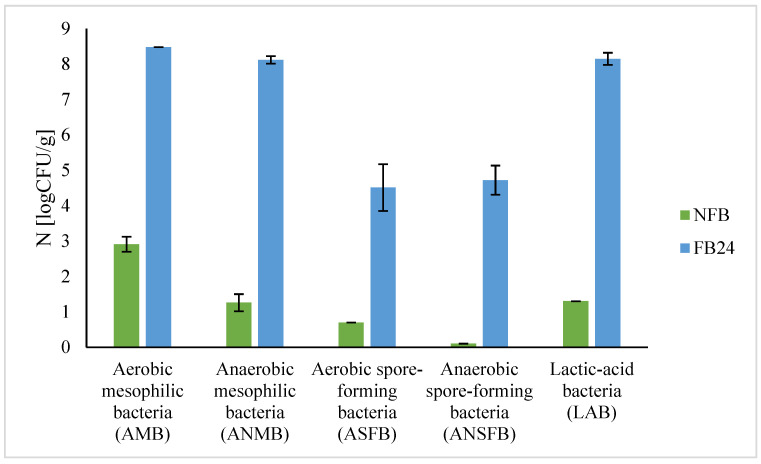
The average number (N [logCFU/g]) of different groups of microorganisms in NFB and FB ± SD.

Microbiological quality of non-fermented and fermented broth is presented in Figure 2 and Table 1, with quantitative and qualitative results of different microorganisms in NFB and FB. NFB contained less than 4 log CFU/g of aerobic mesophilic bacteria (AMB) and also a very small number of anaerobic mesophilic bacteria (ANMB) and lactic acid bacteria, but all spore-forming bacteria were below 1–2 log CFU/g (Figure 2). After 24 h of lactic acid fermentation of spirulina broth, the number of mesophilic bacteria and spore-forming bacteria (aerobic and anaerobic) increased for an average of 3–5 log CFU/g. An increased number of lactic acid bacteria is expected, as *L. plantarum* suspension was added for fermentation.

Results of microbiological examination of *A. platensis* samples prior to and after lactic acid fermentation showed that samples did not contain pathogenic bacteria (Table 1). Yeast and moulds were found neither in NFB nor in FB after 24 h fermentation.

### 3.3. Nutritional Composition

Lactic acid fermentation affected the nutritional value of the microalgal biomass. Proteins are the most abundant nutrient. While there was no significant difference between the average contents of crude proteins in NFB and FB, a significant change could be observed in the ratio between non-protein nitrogen and total nitrogen in favour of non-protein nitrogen content after the fermentation. Significant differences may also be seen in the amount of crude fat, which was lower in the FB. The content of some components also changed after fermentation: lower contents of insoluble fibres and available carbohydrates and higher content of soluble dietary fibres and crude proteins were determined in FB; however, those differences from NFB were not statistically significant (Table 2). Calculated energy values of FB (1390 kJ/100 g DW) and NFB (1425 kJ/100 g DW) were similar.

### 3.4. Total Phenolic Content

**Figure 3:**
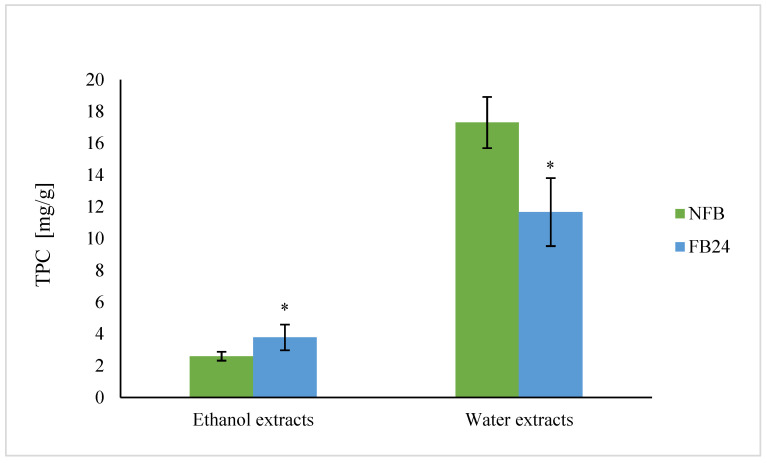
TPC of water and ethanol extracts of NFB and FB. Data represent mean values ± SD. Asterisk (*) indicates statistically significant difference between FB and NFB for particular extract (*p* < 0.05).

Total phenolic content (TPC) of water extracts was in general higher compared with ethanol extracts in FB, as well as in NFB. After fermentation a 33% decrease in TPC was observed in water extracts, while ethanol extracts showed higher content (a 45% increase) compared with NFB (Figure 3).

### 3.5. Antioxidant Activity

#### 3.5.1. In Vitro

**Figure 4:**
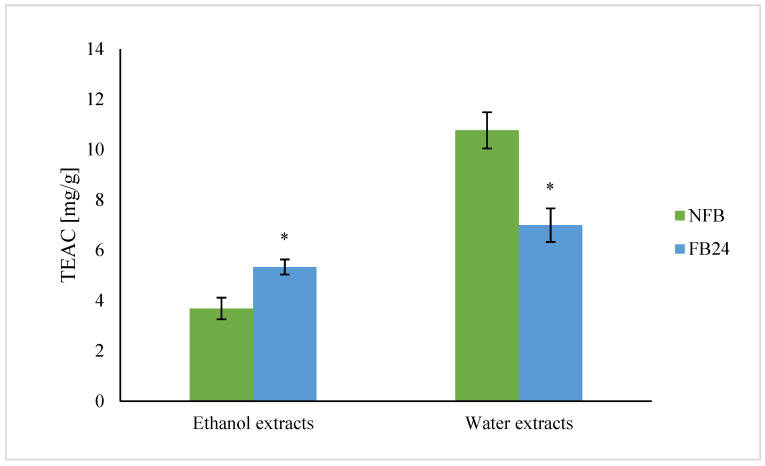
Trolox equivalent antioxidant activity (TEAC) of water and ethanol extracts of non-fermented broth (NFB) and fermented *A. platensis* broth (FB). Data represent mean values ± SD. Asterisk (*) indicates statistically significant difference between FB and NFB for particular extract (*p* < 0.05).

It is shown (Figure 4) that water extracts had higher ability to scavenge DPPH^•^ radicals than ethanol extracts. However, with 24 h fermentation, a 35% decrease in TEAC for water extracts was observed. In the case of ethanol extracts TEAC increased from 3.7 mg/g before fermentation to 5.3 mg/g after fermentation (a 30% increase).

#### 3.5.2. Cellular Antioxidant Activity

**Figure 5:**
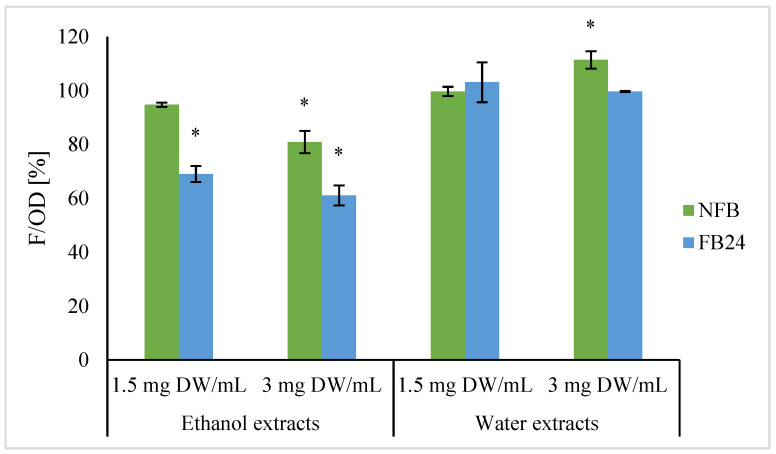
Determination of intracellular oxidation in yeast *Saccharomyces cerevisiae* exposed to water and ethanol extracts of NFB and FB in concentration of 1.5 and 3.0 mg DW/mL. Data represent mean relative values F/OD to the corresponding control set as 100%. Asterisk (*) indicates statistically significant difference between yeast cells treated with NFB or FB extract and control cells (where water or DMSO was added) for particular extract and concentration (*p* < 0.05).

Cellular antioxidant activity was determined using yeast *Saccharomyces cerevisiae* as a model organism. Yeast cells were exposed to water and ethanol extracts of NFB and FB in concentration of 1.5 mg and 3 mg dry extract/mL of yeast suspension. Results are shown in Figure 5.

No changes in intracellular oxidation level were observed when cells were treated with NFB, as well as FB water extracts at a lower concentration, while at a higher concentration a slight increase in oxidation level was observed for NFB water extract.

In contrast, a 20% and 40% decrease in intracellular oxidation was observed when cells were treated with NFB and FB ethanol extracts (3 mg DW/mL), respectively. Similarly, a lower concentration of FB ethanol extracts caused a decrease in cell oxidation level (30%), while no significant changes were observed when cells were exposed to NFB ethanol extract.

### 3.6. Lipid Peroxidation

The cells exposed to FB ethanol extract in a concentration of 1.5 mg DW/mL showed a reduced level of oxidation compared with the control, and lipid peroxidation was further investigated (Table 3). Results show that previous exposure of cells to FB extract decreased the level of oxidative damages caused by the treatment of the cells with menadione.

## 4. Discussion

Lactic acid fermentation of *A. platensis* has already been shown to improve its functional value, including antioxidant activity [5,6]. Thus, the aim of our work was to further evaluate the potential of fermented *A. platensis* biomass to be a component of food products or supplements.

The fermented biomass was investigated in the context of quality, safety, and bioactivity and compared with non-fermented biomass. Thus, determination of nutritional composition and microbiological characterization, as well as antioxidant activity assay, were performed.

Looking at lactic acid fermentation, the most significant changes regarding pH, concentration of lactic acid, and *L. plantarum* growth occurred during the first 24 h of fermentation at 30 °C with *L. plantarum* concentration of 8.5 log CFU/g, lactic acid concentration of 7.4 g/L, and pH value of 5. Niccolai et al. [5] showed that lyophilised *A. platensis* biomass enabled *L. plantarum* growth with a maximal bacterial concentration of 10.6 log CFU/mL at 48 h. Similarly, maximal concentration of lactic acid concentration (3.67 g/L) was reached after 48 h of fermentation at 37 °C and was 2-fold lower compared with our study at 24 h. Thus, in both cases, *A. platensis* biomass was shown to be a suitable substrate for *L. plantarum* growth and fermentation. *A. platensis* cell wall structure is similar to prokaryotic bacteria. It is composed of peptidoglycan. Lactic acid bacteria (LAB) during fermentation degrade cell walls of cyanobacteria by different peptidoglycan hydrolyses. This results in the release and degradation of complex organic molecules of the cells into simpler compounds [6,19], enabling their growth. On the other hand, the release of compounds and/or their metabolism by LAB can mean better functional value of fermented biomass compared with non-fermented. It is known that lactic acid fermentation of food substrates can improve the efficiency of their original bioactive compounds due to their release or transformation by LAB, and thus can enrich the substrate with their metabolites [2]. *A. platensis* has high nutritional value due to its content in proteins, essential amino acids, essential fatty acids, vitamins, minerals, and pigments [20,21], and thus we tested how lactic acid fermentation changed the nutritional composition of spirulina. The content of proteins, lipids, dietary fibres, carbohydrates, and non-protein nitrogen was determined. Analysis showed that during lactic acid fermentation the substrate changed significantly in the content of non-protein nitrogen and crude fat. The non-protein nitrogen in microalgae derives from free amino acids, peptides, amines, amine oxides, and nucleotides [22]. The LAB proteolysis system combines the action of proteinases and peptidases, which efficiently break down proteins into small peptides and amino acids [23]. De Marco Castro et al. [6] found that peptides were released from proteins during lactic acid fermentation of *A. platensis* with *L. plantarum* and that free methionine content was increased as well. Due to protein hydrolysis during mixed fermentation of *A. platensis* by *L. plantarum* and *B. subtilis*, the polypeptide content was increased, proving that fermented *A. platensis* has greater protein bioavailability [23]. Similarly, Yu et al. [7] showed the increased content of amino acids and the ratio of essential amino acids to total amino acids in the fermented *Spirulina* compared with the non-fermented biomass. Additionally, the interest in protein hydrolysates has recently gained importance due to aiding digestive dysfunction and malnutrition [24]. The decrease in crude fat content found in our study is contrary to the findings of Dewi and Amalia [25], where the total fat content in *A. platensis* did not change during fermentation with *L. plantarum.* The discrepancies may be attributed to the different methods used for crude fat extraction and duration of fermentation, which was 24 h in our study, compared with 2–10 days [25]. On the other hand, crude fat contents decreased significantly in studies of bean flours fermentations with *L. plantarum*, where similar procedure for fat determination was used [26,27]. The content of insoluble dietary fibres tends to decrease with lactic acid fermentation. They cannot be digested or absorbed by humans and are insoluble in water. Organic acids and enzymes produced naturally by microorganisms decrease the molecular weight and thus improve their solubility, which may support our findings [28]. Bao et al. [23] found that lactic acid fermentation with *L. plantarum* did not affect the change of soluble polysaccharides in the microalga *A. platensis*. The concentration of polysaccharides is expected to strike a balance between the breakdown of polysaccharides and the formation of bacterial polysaccharides, which may explain the similar values of soluble and total dietary fibres in FB and NFB.

We further checked whether the change in nutritional composition was also reflected in antioxidant activity change. As can be seen from Figure 4, TEAC was higher in water compared with ethanol extracts. This means that hydrophilic antioxidants isolated from *A. platensis* have a higher ability to scavenge DPPH^•^ radical than less polar (ethanol soluble) antioxidants. However, with fermentation, the scavenging ability of water extracts decreased, while ethanol extracts showed higher antioxidant activity. Similar results were obtained for TPC, where the values in water extracts were again higher compared with the ethanol ones, and after fermentation, a decrease in TPC in water extracts and an increase in ethanol extracts were observed. Thus, these results indicate that polyphenols are very likely the most abundantly present antioxidants in *A. platensis*, although it is known that FC reagent used for TPC determination can react also with other nonphenolic compounds present in the extract besides polyphenols, which contribute to higher final content [29]. On the other hand, lactic acid fermentation was shown to be responsible for an increase in DPPH^•^ radical scavenging ability of polyphenols and/or others in 96% ethanol-soluble compounds. These results are in agreement with Curiel et al. [30], who showed that fermentation of medicinal plant myrtle (*Myrtus communis* L.) with *L. plantarum* caused an increase in the concentration of total phenols and antioxidant activity, mostly due to esterase activities of *L. plantarum*. In the presence of this enzyme, the hydrolysis of glycoside bond between a polyphenol and sugar moiety in glycosylated polyphenols occurs. It was already shown that polyphenol aglycones contain multiple hydroxyl groups and hence exhibit a higher antioxidant activity than their glycosides [31]. It was also shown that feruloyl esterases are present in *L. plantarum* strains and are responsible for metabolizing compounds that are abundantly present in fermented plant matrices (e.g., hydroxycinnamoyl esters) [32]. Thus, the increased amount of less polar aglycones (extracted by ethanol) in FB is very likely the reason for the increased antioxidant activity of ethanol extracts after fermentation.

Niccolai et al. [5] performed lactic acid fermentation of *Arthrospira* using *L. plantarum* and showed that after fermentation total phenolic content and antioxidant activity increased significantly (by 320% and 79%, respectively). Similarly, de Marco Castro et al. [6] established that the total phenolic content and antioxidant activity in vitro was enhanced in fermented spirulina compared with untreated biomass. In both cases, water or methanol was used as solvent for the preparation of extracts.

Previous studies [33,34,35,36] have already shown that it is not necessary that antioxidative activity is measured by chemical methods related to antioxidative activity in the cells (CAA). That is, CAA considers bioavailability, cellular uptake, distribution, and metabolism of compounds in the cell. Furthermore, at lower or higher concentrations of compounds, different mechanisms of antioxidative activity could be expressed. That is, antioxidants can directly react with free radicals or inhibit the activity or expression of enzymes related to free radical generation. On the other side, they can enhance the activity or expression of intracellular antioxidant enzymes [37]. We selected yeast *Saccharomyces cerevisiae* in the stationary phase as a model microorganism since such cells are physiologically closest to humans [38,39]. First, cell viability using the CFU method was determined to check any negative effects of extracts on yeast growth, and no changes in viability were observed when yeast cells were treated with water extracts, as well as being treated with ethanol extracts of fermented and non-fermented biomass at concentrations of 1.5 and 3 mg DW/mL (data not shown). Thus, these concentrations were further evaluated to measure antioxidant activity in the cells. In contrast with in vitro antioxidant assay, water extracts did not show a decrease in intracellular oxidation level compared with the control. No difference was observed between non-fermented and fermented broth extracts as well. Although NFB ethanol extracts significantly decreased intracellular oxidation level, an even greater decrease in intracellular oxidation was observed when FB ethanol extracts were used. Petelinc et al. [33] have similarly treated the yeast cells with propolis fractions of different polarities obtained using solid-phase extraction of crude propolis extract and eluted with 30–70% ethanol (EL30-EL70). Among them the greatest decrease in the intracellular oxidation level was observed in the cells treated with less polar EL70 eluate, followed by EL60 and EL50. On the other side, for EL30, they showed a trend of increased intracellular oxidation. Additionally, for the EL70, the cellular uptake of particular phenolic compounds was confirmed to the greatest extent, which might be responsible for the highest antioxidant activity in the cells.

In both cases, in vitro and cellular antioxidant activity of FB ethanol extracts was higher compared with NFB, indicating the role of lactic acid bacteria metabolism in the transformation of *A. platensis* compounds soluble in 96% ethanol and consequently higher bioactivity. Similarly, Li et al. [40] showed the enhancement of CAA of methanol extract of fermented apple juice compared with non-fermented and explained its enhancement as being due to the fact that bacterial metabolism, mainly deglycosylation and degallation activities of apple polyphenol compounds, releases free aglycones where higher number of hydroxyl groups or lower steric hindrance to hydroxyl groups can be found [41].

Further antioxidant activity of FB ethanol extract was confirmed by measuring lipid oxidative damages in yeast cells. Cells that were first exposed to FB ethanol extract and then to menadione as an oxidative stress inductor showed a lower level of oxidative lipid damage, indicating its protective role before oxidative stress compared with the cells exposed only to oxidative stress inductor. Using 96% ethanol as an extraction solvent, more non-polar compounds were extracted, whose further analyses are needed to determine their identity and also to establish which compounds have entered the cells and are responsible for such effect.

The results confirm the nutritional quality and antioxidant activity of fermented biomass, but microbiological safety is another important parameter that has to be evaluated before using it as a component of food products. It is known that lactic acid fermentation can enhance the shelf life of substrates and thus microbiological safety [1]. To evaluate the microbiological safety of fermented *A. platensis* biomass, analysis of different microorganisms was performed (Table 1, Figure 2). Results show that the number of mesophilic bacteria (aerobic, anaerobic) and spore-forming bacteria (aerobic and anaerobic), as well as lactic acid bacteria, increased in FB compared with NFB, which is expected since during fermentation nutrients become more available and bacteria that are already present on the substrate can grow. Further studies about *L. plantarum*’s ability to grow on plate count agar (PCA)—not just on MRS agar (results not shown), which is selective for lactic acid bacteria—explained a higher number of both total aerobic as well as total anaerobic mesophilic bacteria grown on PCA. Similarly, anaerobic spore-forming bacteria were identified in nutraceutical preparations of *A. platensis* for human consumption at a high number (10^5^ CFU/tablet) [42]. Additionally, no presence of pathogenic bacteria, as well as yeasts and moulds in FB after 24 h fermentation, was detected, which is important if we use FB as a food ingredient or supplement. As contamination might occur also during harvest and post-harvest procedures, these results are important for obtaining microbiologically safe *A. platensis* biomass.

## 5. Conclusions

Fermented biomass of *A. platensis* has been shown as a potential source of antioxidants, which showed activity also in the cells, since reduced intracellular ROS level, as well as oxidative damages of lipids, was determined. Compared with non-fermented biomass, the level of non-protein nitrogen increased, indicating higher protein bioavailability, and fat content decreased, while the content of other nutrients remained the same. Additionally, fermented *A. platensis* showed no presence of pathogenic bacteria and has lower pH, indicating enhancement of its shelf life. Therefore, fermented *A. platensis* showed the potential to be used as a nutritional supplement or as an ingredient in food products.

## Figures and Tables

**Table 1:** Pathogenic bacteria, yeast, and moulds in non-fermented (NFB) and fermented *A. platensis* broth (FB).

Microorganisms	N (CFU/g) in NFB	N (CFU/g) in FB
Coliform bacteria	<10	<10
*Escherichia coli*	<100	<100
*Staphylococcus aureus*	<100	<100
*Bacillus cereus*	<100	<100
*Clostridium perfringens*	<100	<100
Yeast	<10	<10
Moulds	<10	<10
*Salmonella* spp. *	Neg. in 10 g	Neg. in 10 g
*L. monocytogenes* *	Neg. in 10 g	Neg. in 10 g

Legend: N, average number [CFU/g], *, qualitative analysis.

**Table 2:** Nutritional composition of non-fermented (NFB) and fermented *A. platensis* broth (FB). Values are expressed as percentage of dry weight. Data represent mean values ± SD.

Component	NFB	FB
Crude protein	46.56 ± 2.43	47.37 ± 1.49
Total ash	12.65 ± 0.34	12.78 ± 0.08
Crude fat	**6.26 ± 0.04**	**6.00 ± 0.01**
Soluble dietary fibres	3.20 ± 0.46	4.02 ± 0.69
Insoluble dietary fibres	19.33 ± 0.70	17.79 ± 0.94
Total dietary fibres	22.53 ± 0.38	21.81 ± 0.72
Available carbohydrates	13.00 ± 0.74	11.05 ± 1.94
Non protein nitrogen/total nitrogen	**24.8% ± 1.5%**	**28.4% ± 1.1%**

Legend: Statistically significantly different values between FB and NFB are written in bold (*p* < 0.05).

**Table 3:** Determination of lipid peroxidation. Data represent mean values of F/OD ± SD. Asterisk (*) indicates a statistically significant difference between yeast cells treated with FB extract followed by menadione and cells treated only with menadione (*p* < 0.05).

Condition	F/OD
Yeast cells	3.37 ± 0.54
Yeast cells + menadione (1 h)	13.8 ± 0.63
Yeast cells + ethanol extract of FB (2 h) + menadione (1 h)	5.71 ± 1.85 *

## Data Availability

Data are contained within the article.
